# The intention and influencing factors of human papillomavirus vaccination among female students in the secondary vocational schools in East China: a cross-sectional study

**DOI:** 10.3389/fpubh.2025.1467546

**Published:** 2025-05-13

**Authors:** Ruihua Zhang, Junhui Du, Zhixiang Zhang

**Affiliations:** ^1^Teaching and Research Office of Midwifery and Surgical Nursing, Changzhou Hygiene Vocational and Technology College, Changzhou, China; ^2^Department of Obstetrics and Gynecology, The First Affiliated Hospital of Ningbo University, Ningbo, China; ^3^Department of Neurology, The Second People’s Hospital of Changzhou, The Third Affiliated Hospital of Nanjing Medical University, Changzhou, China

**Keywords:** secondary vocational school, human papilloma virus, vaccination, intention, influencing factor

## Abstract

**Objective:**

This study aimed to explore the intention and influencing factors toward HPV vaccination among female students in secondary vocational schools in East China.

**Methods:**

A cross-sectional study was conducted among 500 female students aged 16–20 years from four secondary vocational schools in East China from September to December 2023. The information, including HPV and HPV vaccine–related knowledge questionnaire, family characteristics, attitudes toward premarital sex, information sources, and HPV vaccination or appointment status, was collected through an online questionnaire survey. According to the vaccination or appointment status, 421 participants who met the inclusion criteria were divided into two groups. The relationship between different factors and HPV vaccination intention was assessed using univariate and multivariate logistic analyses.

**Results:**

Out of 421 participants, 286 (67.9%) were in the nonappointment group, while 135 (32.1%) were in the appointment/vaccination group. There was no significant difference in HPV and HPV vaccine–related knowledge scores and attitudes toward premarital sex between the two groups (*p* > 0.05). The factors such as grade level, family characteristics (including family’s residence, parents’ highest education level, monthly living expenses, and only child), and information sources showed significant differences between the two groups (*p* < 0.05). After multivariate logistic regression analysis (analyzing one characteristic at a time and adjusting all other characteristics as confounders), factors such as higher grades (grade 3 [odds ratio, OR = 0.39; 95% confidence interval, CI: 0.22–0.68], grade 4 [OR = 0.03; 95% CI: 0.02–0.07]), family’s residence in urban areas (OR = 1.94; 95% CI: 1.1–3.41), monthly living expenses ≥2000 yuan (OR = 2.19; 95% CI: 1.08–4.47), only child (OR = 2; 95% CI: 1.2–3.36), and information sources from family or friends (OR = 2.34; 95% CI: 1.18–4.62), were independent influencing factors of HPV vaccination intention (all *p* < 0.05).

**Conclusion:**

The HPV vaccination policy of the local government significantly impacts accination intention. Meanwhile, family factors are independent influencing factors on HPV vaccination intention. When promoting HPV vaccination, secondary vocational schools in East China should focus on senior female students from rural areas, low-income families, and families with multiple children.

## 1 Introduction

In the past 20 years, the number of new cases and deaths from cervical cancer worldwide has been continuously increasing. According to the International Agency for Research on Cancer (IARC) 2022 global cancer burden data (covering 36 types of cancer in 185 countries/regions worldwide), cervical cancer has become the leading tumor among women in 25 countries and the leading cause of cancer death among women in 37 countries (1). Similarly, the incidence of cervical cancer in China also continues to rise predominantly in the youth. Its incidence in 2022 was 11.25/10^5^, of which 99.7% of cases were related to human papillomavirus (HPV) infection (2, 3). HPV vaccination can significantly reduce HPV infection in women, thereby reducing the risk of cervical cancer (4, 5). The Cervical Cancer Elimination Initiative (CCEI) released by the World Health Organization (WHO) on November 17, 2020, requires 90% of girls to be fully vaccinated against HPV before the age of 15 years by 2030 (6, 7). However, the HPV vaccination rate for girls aged 9–14 years is still <5% in China (8).

HPV vaccine is not currently included in the National Immunization Program (NIP) in China, with its vaccination remaining voluntary and self-funded in most areas of the Chinese Mainland (9). Therefore, studying the intention and influencing factors of HPV vaccination among girls can provide a reference for formulating strategies to promote vaccination. Although there are differences in the study population and observation indicators, factors such as HPV and HPV vaccine–related knowledge, family economic status, attitudes toward premarital sex, and vaccine accessibility may affect vaccination intention (10–14).

In East China, although female students in secondary vocational schools are aged >15 years, most of them have not had sexual intercourse and are still can be vaccinated. On the other hand, due to limited research on this particular group, little is known about their HPV vaccination intention and influencing factors.

Therefore, this study aimed to explore the intention and influencing factors toward HPV vaccination among female students in secondary vocational schools in East China, thus providing a reference for developing vaccination strategies targeting this specific group.

## 2 Methods

### 2.1 Study participants

Based on a 95% confidence level, a 5% margin of error, and an estimated response rate of 80% (considering that students in school may have higher cooperation), a minimum sample size of 453 female students was required for this study.

This study employed a multi-stage sampling method for participant recruitment. In the first stage, we randomly selected 4 out of 11 secondary vocational schools located in Changzhou, Jiangsu Province, eastern China, using schools as sampling units. The selected schools were Changzhou Vocational and Technical College of Tourism and Commerce, Changzhou Higher Vocational and Technical College, Changzhou Hygiene Vocational and Technology College, and Changzhou Art Vocational and Technical College. The number of female students (grades 1–4) in these four schools was 1,267, 1,412, 2,214, and 1,131 respectively, totaling 6,024. In the second stage, we randomly selected 105, 117, 184, and 94 female students from the aforementioned schools in equal proportions (8.3%), totaling 500 individuals.

Individuals were included if they could clearly express their thoughts, if both they and their legal guardians signed informed consent, and if they were willing to participate in online surveys. Individuals were excluded if they failed to complete the online survey within 12 h, if their questionnaire answers were incomplete, if they answered that they had not heard of HPV, and if their questionnaire completion time fell below the 2.5th percentile or above the 97.5th percentile. Based on the sample size calculation, the final number of participants required for this study was at least 385.

The Ethics Committee of First Affiliated Hospital of Ningbo University approved this study (approval number: XJS20231212), which was conducted according to the Declaration of Helsinki. We obtained informed consent from 480 individuals.

### 2.2 Questionnaire design

We systematically analyzed the HPV vaccination intention survey questionnaires used in published studies over the past 5 years (10–14) and subsequently designed the initial draft of the questionnaire for this study. Then, the initial draft was discussed and revised by an expert group (comprising five deputy directors or chief physicians of gynecology from tertiary hospitals) to form a formal questionnaire. In the final formal questionnaire, the item-level content validity index (I-CVI) of all items was 1, and the average value of the scale-level content validity index (S-CVI) is also 1. Afterward, we conducted a preliminary survey of 50 female students in grades 1–4 at the author’s affiliated school. The Cronbach’s alpha coefficient of the formal questionnaire was 0.927, indicating acceptable internal consistency for research purposes.

### 2.3 Questionnaire survey and data collection

The formal questionnaire included the following items: whether you have heard of HPV, grade level, family’s residence, parental education level, monthly living expenses, whether you are an only child, attitudes toward premarital sex, HPV-related knowledge, HPV vaccine–related knowledge, information sources, and HPV vaccination or appointment status. Among them, the total score was calculated for HPV and HPV vaccine–related knowledge (1 point for each correct answer).

This study used the Tencent Questionnaire Star app to distribute survey questionnaires online in December 2023, with each IP address restricted to one response per questionnaire. The system could automatically record the time and duration of questionnaire answering. All participants were required to complete the questionnaire within 12 h.

### 2.4 Statistical analysis

All participants were divided into two groups based on HPV vaccination or appointment status: nonappointment or appointment/vaccination groups. Data were presented as mean ± standard deviation (SD) for continuous variables and as absolute with percentage for categorical variables.

In the analysis of the basic characteristics, HPV, and HPV vaccine–related knowledge scores, statistical differences between the two groups were assessed using a t-test for continuous variables and a chi-square for categorical variables. Items with a *p* < 0.05 were included in univariate logistic regression analysis to analyze their impacts on HPV vaccination intention. Items with *p* < 0.05 in the univariate analysis of HPV vaccination intention were individually subjected to multivariate logistic regression analysis, with each item analyzed one at a time while adjusting for all other items as potential confounders, to assess their independent impact.

The significance level was set at *p* < 0.05 (two-tailed). All analyses were performed using R Statistical Software (version 4.2.2, http://www.R-project.org, the R Foundation) and the Free Statistics analysis platform (version 1.8).

## 3 Results

### 3.1 Basic information of questionnaire survey

A total of 480 female students received a questionnaire survey, among which 11 incompletely answered the questionnaire, and 469 (97.7%) completed the questionnaire. Among the complete questionnaires, 48 questionnaires (of which 25 [5.3%] participants answered that they had never heard of HPV, while 23 girls answered outside the range of 2.5 to 97.5%) were excluded. The final 421 participants’ questionnaires were included in the statistical analysis, including 286 (67.9%) in the nonappointment group and 135 (32.1%) in the appointment/vaccination group.

### 3.2 Comparison of basic characteristics between the two groups

Table 1 shows the basic characteristics of the study participants. There was no significant difference in attitudes toward premarital sex between the two groups (*p* > 0.05). The items such as grade level, family’s residence, parents’ highest education level, monthly living expenses, information sources, and being an only child showed significant differences between the two groups (*p* < 0.05).

**Table 1:** Comparison of basic characteristics between the two groups (n = 421).

Variable	Total		HPV vaccination status				*p*	*χ^2^*
			Nonappointment group		Appointment/vaccination group			
	*n*	*%*	*n*	*%*	*n*	*%*		
Grade lever							< 0.001	111.925
Grade 1	94	22.3	36	12.6	58	43		
Grade 2	39	9.3	16	5.6	23	17		
Grade 3	109	25.9	65	22.7	44	32.6		
Grade 4	179	42.5	169	59.1	10	7.4		
Family’s residence							< 0.001	11.018
Rural areas	167	39.7	129	45.1	38	28.1		
Urban areas	254	60.3	157	54.9	97	71.9		
Parental highest education							0.002	13.001
Elementary or less	95	22.6	77	26.9	18	13.3		
Secondary school or college	191	45.4	130	45.5	61	45.2		
Bachelor’s and above	135	32.1	79	27.6	56	41.5		
Monthly living expenses							0.011	8.95
<1,000 yuan	87	20.7	64	22.4	23	17		
1,000–2,000 yuan	274	65.1	191	66.8	83	61.5		
≥2,000 yuan	60	14.3	31	10.8	29	21.5		
Only child							< 0.001	13.417
No	223	53.0	169	59.1	54	40		
Yes	198	47.0	117	40.9	81	60		
Attitudes toward premarital sex							0.095	2.787
Nonacceptance	306	72.7	215	75.2	91	67.4		
Acceptance	115	27.3	71	24.8	44	32.6		
Information source							< 0.001	16.632
School education	216	51.3	155	54.2	61	45.2		
Nonmedical journals or media	95	22.6	66	23.1	29	21.5		
Medical journals or media	44	10.5	34	11.9	10	7.4		
Family or friends	66	15.7	31	10.8	35	25.9		

### 3.3 Comparison of HPV and HPV vaccine–related knowledge scores between the two groups

Table 2 demonstrates the HPV and HPV vaccine–related knowledge scores of the study participants. HPV and HPV vaccine–related knowledge scores were not significantly different between the two groups (*p* > 0.05).

**Table 2:** Comparison of HPV and HPV vaccine–related knowledge scores between the two groups.

Knowledge scores	Nonappointment group	Appointment/vaccination group	*p*	*t*
	Mean ± SD	Mean ± SD		
HPV-related knowledge scores	16.1 ± 2.1	16.5 ± 1.8	0.080	3.074
HPV vaccine–related knowledge scores	8.0 ± 1.7	7.9 ± 1.8	0.720	0.129

### 3.4 Univariate logistic regression analysis of the influence of basic characteristics on HPV vaccination intention

Table 3 provides the results of the univariate logistic regression analysis of the influence of basic characteristics on HPV vaccination intention. Compared to grade 1, HPV vaccination intention was lower in grade 3 (odds ratio [OR] = 0.42; 95% CI: 0.24–0.74) and grade 4 (OR = 0.04; 95% CI: 0.22–0.08), while there was no significant difference in grade 2 (OR = 0.89; 95% CI: 0.42–1.91). The characteristics such as family’s residence in urban areas (OR = 2.1; 95% CI: 1.35–3.26), higher parental education level (secondary school/college [OR = 2.01; 95% CI: 1.11–3.64], bachelor’s degree or above [OR = 3.03; 95% CI: 1.64–5.62]), monthly living expenses ≥ 2000 yuan (OR = 2.6; 95% CI: 1.3–5.22), being an only child (OR = 2.17; 95% CI: 1.43–3.29), and information source from family or friends (OR = 2.87; 95% CI: 1.63–5.06) contributed to a higher HPV vaccination intention.

**Table 3:** Univariate logistic regression analysis of basic characteristics.

Variable	*OR* (95%*CI*)	*p*
Grade lever		
Grade 1	1 (reference)	
Grade 2	0.89 (0.42–1.91)	0.769
Grade 3	0.42 (0.24–0.74)	0.003
Grade 4	0.04 (0.02–0.08)	< 0.001
Family’s residence		
Rural areas	1 (reference)	
Urban areas	2.1 (1.35–3.26)	0.001
Parental highest education		
Elementary or less	1 (reference)	
Secondary school or college	2.01 (1.11–3.64)	0.022
Bachelor’s and above	3.03 (1.64–5.62)	< 0.001
Monthly living expenses		
<1,000 yuan	1(reference)	
1,000–2,000 yuan	1.21 (0.7–2.08)	0.492
≥2,000 yuan	2.6 (1.3–5.22)	0.007
Only child		
No	1(reference)	
Yes	2.17 (1.43–3.29)	< 0.001
Source of information		
School education	1(reference)	
Nonmedical journals or media	1.12 (0.66–1.89)	0.682
Medical journals or media	0.75 (0.35–1.61)	0.455
Family or friends	2.87 (1.63–5.06)	< 0.001

### 3.5 Multivariate logistic regression analysis of the independent impact of basic characteristics on HPV vaccination intention

Participant characteristics with *p* < 0.05 in the univariate analysis on HPV vaccination intention were individually subjected to multivariate logistic regression analysis (analyzing one characteristic at a time and adjusting all other characteristics as confounders). Characteristics such as higher grade (grade 3 [OR = 0.39; 95% CI: 0.22–0.68], grade 4 [OR = 0.03; 95% CI: 0.02–0.07]), family’s residence in urban areas (OR = 1.94; 95% CI: 1.1–3.41), monthly living expenses ≥2000 yuan (OR = 2.19; 95% CI: 1.08–4.47), being an only child (OR = 2; 95% CI: 1.2–3.36), and information sources from family or friends (OR = 2.34; 95% CI: 1.18–4.62) were the independent influencing factors of HPV vaccination intention (Table 4).

**Table 4:** Multivariate logistic regression analysis of basic characteristics.

Variable	*OR* (95%*CI*)	*p*
Grade lever		
Grade 1–2	1 (reference)	
Grade 3	0.39 (0.22–0.68)	0.001
Grade 4	0.03 (0.02–0.07)	<0.001
Family’s residence		
Rural areas	1 (reference)	
Urban areas	1.94 (1.1–3.41)	0.021
Parental highest education		
Elementary or less	1 (reference)	
Secondary school or college	1.55 (0.77–3.12)	0.216
Bachelor’s and above	1.48 (0.69–3.19)	0.312
Monthly living expenses ≥2,000 yuan		
No	1 (reference)	
Yes	2.19 (1.08–4.47)	0.030
Only child		
No	1 (reference)	
Yes	2 (1.2–3.36)	0.008
Source of information is family or friends		
No	1 (reference)	
Yes	2.34 (1.18–4.62)	0.015

## 4 Discussion

This cross-sectional study investigated the intention and influencing factors of HPV vaccination among female students in secondary vocational schools in East China. We found that grade level was a strong independent influencing factor in the study population, with lower vaccination intention observed in grades 3–4. Additionally, certain family characteristics (including family residence in urban areas, monthly living expenses ≥2000 yuan, and being an only child) and information sources from family or friends were independent influencing factors for higher HPV vaccination intention. However, the level of HPV and HPV vaccine–related knowledge was not related to vaccination intention.

The HPV vaccine was approved for the first time in the Chinese Mainland in 2016. However, three studies conducted in the Chinese Mainland in 2021 revealed that the vaccination rate of HPV vaccine was only approximately 3%, while the awareness rate of HPV was only 33–59.89% (15–17). Developed countries in Europe and America had higher awareness of HPV and HPV vaccines during the same period (18, 19). Multiple studies showed that awareness of HPV and HPV vaccines is closely related to vaccination rates (13, 20). In recent years, with the rise of the Internet and We Media, HPV and HPV vaccine-related knowledge in the Chinese Mainland has been rapidly popularized at the social level (21). In this study, only 5.3% of female students reported not having heard of HPV. The lack of an association between HPV and HPV vaccine–related knowledge and vaccination intention in this study might be attributed to the aforementioned factors.

Among 194 member countries of the WHO, 66.5% have included the HPV vaccine in their National Immunization Program (NIP) (22). Although China has not yet included the HPV vaccine in its NIP (23), some regions in East China, including 4 out of 13 cities in Jiangsu Province, started promoting free HPV vaccination among female students aged 9–15 years in 2021 (24, 25). In this study, the majority of female students in grades 3–4 were aged >15 years in 2021 and not within the scope of local government HPV vaccination promotion, explaining the strong independent influence of grade level on HPV vaccination intention. This underscores the importance of government vaccine policies and highlights that the focus of HPV vaccine promotion work was on female students who were aged >15 years in 2021.

Currently, HPV vaccination in most eastern regions of China still requires self-payment, with a relatively expensive vaccine cost (26, 27). Therefore, in the absence of government promotion of free vaccination, whether female students are vaccinated against HPV is generally decided by their parents. We also observed a significant impact of family factors on HPV vaccination intention in this study. Monthly living expenses ≥2000 yuan reflect a good family financial situation, and only children will receive more attention from their parents. The family source of the HPV-related information also reflects parents’ concerns about HPV. There are more HPV vaccination institutions in urban areas compared to rural areas. Hence, we should pay more attention to female students from rural areas, low-income families, and families with multiple children. On January 5, 2023, the Chinese government released the “Action Plan for Accelerating the Elimination of Cervical Cancer (2023–2030),” which requires the promotion of HPV vaccination (28). We speculate that the influence of family factors on HPV vaccination intention will gradually diminish as the plan progresses, and we plan to verify this in subsequent studies.

This study had several limitations. First, due to regional differences in HPV vaccination in China (29), the conclusions of this study were derived from the female student population in vocational schools in East China and could not be extended to other regions or all female populations in China. Second, we used a total score when analyzing HPV and HPV vaccine–related knowledge; therefore, we are uncertain whether certain specific knowledge points have an impact on vaccination intention. Third, we did not anticipate the significant differences in HPV vaccination rates among different grades during the research design; therefore, we did not differentiate between grades during random enrollment, resulting in an uneven number of participants among different grades. This might have increased the bias of the results to some extent. Lastly, all participants in this study were female; however, male vaccination with the HPV vaccine can also reduce the risk of HPV infection (30). We will conduct corresponding research on male students in secondary vocational schools in the future.

## 5 Conclusion

This study found that the HPV vaccination policy of the local government has a significant impact on vaccination intention. Meanwhile, family factors are an independent influencing factor for HPV vaccination willingness. Secondary vocational schools in eastern China should focus on senior female students from rural areas, low-income families, and families with multiple children when promoting HPV vaccination.

## Data Availability

The datasets presented in this study can be found in online repositories. The names of the repository/repositories and accession number(s) can be found in the article/Supplementary material.
