# Supplementary material for: The intention and influencing factors of human papillomavirus vaccination among female students in the secondary vocational schools in East China: a cross-sectional study
Source: Front Public Health. 2025 May 13;13:1467546. doi: 10.3389/fpubh.2025.1467546 (PMC12106446; doi:10.3389/fpubh.2025.1467546)
Supplement: Supplementary file 1 [file Table_1.docx]

**Survey Questionnaire on** **Human Papilloma Virus Vaccination Willingness and Influencing Factors among Female Students in Secondary Vocational Schools**

This questionnaire consists of four parts. Please read each question carefully and choose the appropriate option based on your personal knowledge and experience.

| **Part I: Basic Information** | | |
| --- | --- | --- |
| 1 | grade lever | ○1 ○2 ○3 ○4 |
| 2 | family’s residence | ○rural areas ○urban areas |
| 3 | Parental highest education | ○elementary or less  ○secondary school or college  ○bachelor’s and above |
| 4 | monthly living expenses | ○<1000 yuan  ○1000元-2000 yuan  ○≥2000 yuan |
| 5 | whether you are an only child | ○yes ○no |
| 6 | attitudes toward premarital sex | ○acceptance ○nonacceptance |
| 7 | whether you have heard of HPV | ○yes ○no |
| 8 | source of information | ○school education ○nonmedical journals or media ○medical journals or media ○family or friends |
| **Part II: HPV-related knowledge** | | |
| 9 | HPV infection is very rare | ○yes ○no |
| 10 | HPV infection always presents obvious symptoms | ○yes ○no |
| 11 | HPV infection can cause cervical cancer | ○yes ○no |
| 12 | HPV can be transmitted through contact with the skin or mucous membranes of the reproductive organs | ○yes ○no |
| 13 | There are various types of HPVs | ○yes ○no |
| 14 | HPV can be transmitted through sexual activity | ○yes ○no |
| 15 | HPV can cause genital warts | ○yes ○no |
| 16 | Men will not be infected with HPV | ○yes ○no |
| 17 | Using condoms can reduce the chance of HPV transmission | ○yes ○no |
| 18 | HPV can be cured with antibiotics | ○yes ○no |
| 19 | Having too many sexual partners increases the risk of HPV infection | ○yes ○no |
| 20 | HPV infection usually does not require any treatment | ○yes ○no |
| 21 | Most sexually active individuals will be infected with HPV at some point in their lives | ○yes ○no |
| 22 | A person may be unknowingly infected with HPV for many years | ○yes ○no |
| 23 | Premature sexual activity increases the risk of HPV infection | ○yes ○no |
| 24 | HPV infection can lead to anal cancer | ○yes ○no |
| 25 | HPV is a bacterial infection | ○yes ○no |
| 26 | HPV can be transmitted through oral sex | ○yes ○no |
| 27 | HPV infection can cause herpes | ○yes ○no |
| 28 | HPV infection can be transmitted through anal intercourse | ○yes ○no |
| 29 | HPV infection always leads to health issues | ○yes ○no |
| 30 | HPV infection can lead to oral cancer | ○yes ○no |
| 31 | Asymptomatic individuals do not transmit HPV | ○yes ○no |
| **Part III: HPV vaccine- related knowledge** | | |
| 32 | HPV vaccine only requires one shot | ○yes ○no |
| 33 | The HPV vaccine can prevent all sexually transmitted diseases | ○yes ○no |
| 34 | It is best to get the HPV vaccine before the first sexual intercourse | ○yes ○no |
| 35 | If you get the HPV vaccine, you won't get cervical cancer | ○yes ○no |
| 36 | HPV vaccine can prevent most cervical cancers | ○yes ○no |
| 37 | HPV vaccine can prevent genital warts | ○yes ○no |
| 38 | Girls who have received the HPV vaccine do not need to undergo cervical cancer screening as they grow up | ○yes ○no |
| 39 | The HPV vaccine can protect you from various types of HPV infections | ○yes ○no |
| 40 | Vaccination with HPV can cure HPV infection | ○yes ○no |
| 41 | Knowing the recommended age for female HPV vaccination in China at present | ○yes ○no |
| **Part IV: Willingness to receive HPV vaccine** | | |
| 42 | Have you received/made an appointment for the HPV vaccine | ○appointment or vaccination ○nonappointment |
| 43 | Why are you currently unwilling to get vaccinated | ○Believing oneself not to be infected with HPV  ○Believing that the HPV vaccine is not effective for oneself  ○Fear of injections or side effects  ○Economic reasons  ○Family members disagree  ○The doctor thinks I'm not suitable for playing  ○I don't know where to get vaccinated  ○Other reasons |
